# Design and methods for a randomized clinical trial treating comorbid obesity and major depressive disorder

**DOI:** 10.1186/1471-244X-8-77

**Published:** 2008-09-15

**Authors:** Kristin L Schneider, Jamie S Bodenlos, Yunsheng Ma, Barbara Olendzki, Jessica Oleski, Philip Merriam, Sybil Crawford, Ira S Ockene, Sherry L Pagoto

**Affiliations:** 1 Department of Medicine, Division of Preventive and Behavioral Medicine, University of Massachusetts Medical School, 55 Lake Avenue North, Worcester, MA, USA; 2 Department of Cardiovascular Medicine, University of Massachusetts Medical School, 55 Lake Avenue North, Worcester, MA, USA

## Abstract

**Background:**

Obesity is often comorbid with depression and individuals with this comorbidity fare worse in behavioral weight loss treatment. Treating depression directly prior to behavioral weight loss treatment might bolster weight loss outcomes in this population, but this has not yet been tested in a randomized clinical trial.

**Methods and design:**

This randomized clinical trial will examine whether behavior therapy for depression administered prior to standard weight loss treatment produces greater weight loss than standard weight loss treatment alone. Obese women with major depressive disorder (N = 174) will be recruited from primary care clinics and the community and randomly assigned to one of the two treatment conditions. Treatment will last 2 years, and will include a 6-month intensive treatment phase followed by an 18-month maintenance phase. Follow-up assessment will occur at 6-months and 1- and 2 years following randomization. The primary outcome is weight loss. The study was designed to provide 90% power for detecting a weight change difference between conditions of 3.1 kg (standard deviation of 5.5 kg) at 1-year assuming a 25% rate of loss to follow-up. Secondary outcomes include depression, physical activity, dietary intake, psychosocial variables and cardiovascular risk factors. Potential mediators (e.g., adherence, depression, physical activity and caloric intake) of the intervention effect on weight change will also be examined.

**Discussion:**

Treating depression before administering intensive health behavior interventions could potentially boost the impact on both mental and physical health outcomes.

**Trial registration:**

NCT00572520

## Background

Obesity is a serious public health threat in the United States with 32.2% of the population affected [1]. Links between obesity and depression have been observed in clinical and epidemiological studies, particularly among women. In one clinic sample, 34% of treatment-seeking obese women were found to have major depressive disorder (MDD) [2]. This is concerning because depression is associated with worse outcomes in behavioral weight loss treatment [2]. However, the vast majority of randomized clinical trials testing obesity treatments either exclude individuals who are clinically depressed [3,4] or do not assess depression status [5-9]; thus evidence for the efficacy of behavioral weight loss treatments for depressed, obese individuals is lacking. Given the concurrence of depression with obesity and the difficulty depressed individuals have losing weight, behavioral weight loss treatments that address the unique needs of this population are needed.

A comorbid relationship between obesity and depression suggests that the two conditions may have related mechanistic pathways, which could have implications for treatment. Obesity is associated with anhedonia [10], a reduced capacity to experience pleasure, which is also a primary symptom of MDD [11,12]. Obese women appear less able to elicit pleasure from ordinarily pleasurable experiences compared to their lean counterparts [10]. When comorbid with depression, obesity may be in part attributable to maladaptive mood regulatory habits involving overeating and inactivity. Targeting weight loss through dieting could thwart this mood regulatory process by facilitating feelings of deprivation, causing compensatory overconsumption and worsened depressive symptoms. Treatments for depression in the context of obesity should directly address the links between mood, eating, and active versus sedentary behaviors, but traditional behavioral weight loss treatments were not necessarily designed to target this combination of issues, and neither were depression psychotherapies. However, behavioral treatments for depression that address maladaptive mood regulatory habits may be helpful in the context of obesity, to the extent that eating behavior and inactivity can be specifically targeted.

A treatment emanating from behavioral theory, behavioral activation [12,13], aims to increase exposure to the positive consequences of healthy behavior, for the purpose of increasing engagement in healthy behaviors, reducing engagement in depressive behaviors and decreasing avoidance behavior[13,14]. The focus of behavioral activation on increasing healthy activity and eliminating avoidance is appropriate to the treatment goals of depression and obesity. Weight loss could be facilitated by increasing an individual's arsenal of mood regulatory behaviors to break an over reliance on eating for coping with negative moods, and to gradually adopt more physically active coping behaviors. Avoidance by obese patients can take the form of excessive eating or prolonged sedentary activities that prevent engagement in other more reinforcing and active aspects of life. Increasing coping behaviors that are physically active or incompatible with eating may help to reduce avoidance behavior, depressive symptoms, and ultimately weight.

The efficacy of behavioral activation for depression has been demonstrated in a variety of populations and settings [15-18]. In a pilot study, our group demonstrated reductions in depression, good treatment adherence, and high treatment satisfaction for an intervention that delivered behavioral activation simultaneously with a brief nutrition counseling intervention [19]. However, in that study, average weight loss was modest and the greatest losses were observed in the latter weeks of treatment, after depressive symptoms had improved. Improving depressive symptoms prior to an intensive dietary intervention might be a more effective sequence of treatment delivery for weight loss outcomes in women with depression.

### Research Goals

The present study aims to compare the efficacy of a sequential two-part treatment, behavioral activation for depression followed by standard weight loss treatment (BA), to standard weight loss treatment that is not accompanied by depression counseling (ST) on weight change in depressed, obese women. The primary hypothesis is that participants in the BA condition will achieve greater weight loss than participants in the ST condition at 6-months and 1- and 2-year follow-up. Secondary research goals include comparing groups on change in depression, physical activity, daily caloric intake, psychosocial variables (emotional eating, quality of life), and cardiovascular risk factors (blood pressure, serum lipids, C-reactive protein and waist circumference) at 6-months and 1- and 2-year follow-up. Additionally, potential mediators (treatment adherence, depression, physical activity, caloric intake) of the intervention effect on weight change will be tested.

## Methods

### Study Design

This is a randomized controlled trial where obese participants with MDD are randomized to one of two treatment conditions: BA or ST. All procedures and material were approved by the University of Massachusetts Medical School's Internal Review Board.

### Eligibility Criteria

Women between the ages of 21 and 65 years old who meet criteria for obesity and MDD will be recruited. Table 1 summarizes the inclusion and exclusion criteria. Exclusion criteria were derived to: 1) minimize adverse effects of the intervention (e.g., physical limitations, does not receive physician clearance); 2) decrease error associated with the primary outcomes (e.g., taking weight loss medication or medications that influence weight); 3) prevent missing data (e.g., plans to move, no telephone) and 4) prevent participants who may require more intensive or more appropriate psychological intervention from enrolling in the study (e.g., active suicidal ideation, bipolar disorder, psychotic disorder). Participants who have been taking antidepressant medications (ADM) for more than 3 months and have no plans to change the regimen will be eligible. While participants who are on ADM will not be excluded, those in psychotherapy will be excluded because of potential conflicting therapeutic goals and possible contamination in the no therapy condition.

**Table 1:** Participant eligibility and exclusion criteria.

**Eligibility Criteria**	
	Female
	Major depressive disorder
	Age: 21–65 years
	Body mass index: 30–40 kg/m^2^
	
**Exclusion Criteria**	
	Unable/unwilling to provide informed consent
	Plans to move out of area during study period
	Smoker
	Bipolar, psychotic, or post-traumatic stress disorder, bulimia
	Severe depression: HDRS > 24 or BDI-II >29
	Type 1 or 2 diabetes
	Had or plans to have bariatric surgery
	No telephone
	Unable to walk unaided or cannot walk 1/4 mile without stopping
	Does not have written clearance from primary care provider for study participation
	Has a medical condition which precludes dietary changes
	Has medical conditions likely to limit lifespan
	Taking prescription weight loss medications
	Initiated anti-depressant medication within the previous 6 weeks
	Active suicidal ideation
	Psychiatric hospitalization in the past 12 months
	Currently in psychotherapy
	Taking mood stabilizers, anti-psychotic medication or medications known to affect appetite and/or weight

Note. HRSD = Hamilton Rating Scale for Depression; BDI-II = Beck Depression Inventory-II.

### Recruitment

A computerized Patient Health Care Information System used in the University of Massachusetts Memorial (UMM) primary care clinics will identify participants who are potentially eligible using age, female gender, and body mass index ranges. Candidates will be contacted via an introductory letter signed by their primary care physician and the principal investigator (PI). Candidates will be given the opportunity to opt out by phone, or be phoned for an initial telephone screening two weeks after receiving the letter. Participants will also be recruited from the community via media outlets, the medical center intranet, and community flyers. Minority populations will be targeted by distributing flyers to businesses and health clinics that serve predominately minority populations and using advertisements in media outlets that have a large minority audience base. Free seminars about women's health will also be conducted at local businesses, community organizations, and churches to facilitate recruitment.

### Screening Process and Informed Consent

Initial eligibility will be assessed by phone and pre-eligible participants will be invited to the study visit for a screening appointment where informed consent will be obtained and eligibility will be further assessed. The Structured Clinical Interview for the Diagnostic and Statistical Manual-IV [20] will be administered to confirm the diagnosis of MDD. Eligible participants will then complete a baseline assessment visit, for which they will receive $50 (see Table 2 for the list of screening and baseline measures). Those not qualifying will receive referrals to weight loss and/or depression treatment, when applicable.

**Table 2:** List of measures and frequency.

	Screening	Baseline	Month 6	Year 1	Year 2
Structured Clinical Interview for DSM-IV	X		X	X	X
Hamilton Rating Scale for Depression	X	X	X	X	X
Beck Depression Inventory	X	X	X	X	X
Medical History Questionnaire	X		X	X	X
Brief Medication Questionnaire	X		X	X	X
Body weight	X	X	X	X	X
Height	X				
Waist and hip circumference		X	X	X	X
Blood pressure		X	X	X	X
Fasting blood: Cholesterol, LDL, HDL, triglycerides, C-reactive protein		X	X	X	X
Three 24-hour dietary recalls		X	X	X	X
Three 24-hour physical activity recalls		X	X	X	X
Fawcett-Clark Hedonic Capacity		X	X	X	X
Short-form 36 Health Survey		X	X	X	X
Dutch Eating Behavior Questionnaire		X	X	X	X
Paffenbarger Physical Activity Questionnaire		X	X	X	X
Environmental Reward Observational Scale		X	X	X	X

Note. DSM-IV = Diagnostic and Statistical Manual of Mental Disorders, Fourth Edition; LDL = low-density lipoprotein; HDL = high-density lipoprotein

### Randomization

After completing the baseline visit and 24 hour diet and physical activity recalls, participants will be randomized to the BA or ST condition. Participants will be stratified into 4 strata of possible combinations of ADM (2: present, absent) × MDD severity (2: Hamilton Rating Scale for Depression (HRSD); [21]: 13–18, 19–24) at baseline. Within each strata, participants will be randomized to the two conditions in randomly permuted blocks of size 3 and 6 using the ralloc program in Stata [22]. This will ensure that the distribution of ADM status and MDD severity are approximately similar between two study conditions.

### Outcomes and Study Measures

The main study outcome will be body weight measured using the digital Tronix scale, with the participant wearing only light clothing and no shoes. Body weight will be measured at every study visit, but analyses will focus primarily on change in body weight from baseline to the 6-months and 1- and 2-year follow-up.

Secondary outcomes include depression (HRSD and Beck Depression Inventory-II (BDI-II); [23]), physical activity and dietary intake, other psychosocial variables (emotional eating, quality of life), and cardiovascular risk factors (blood pressures, serum lipids, C-reactive protein) at 6-months and 1- and 2-year follow-up.

Treatment adherence data will also be collected at all assessment time points to examine possible mechanisms of the effect of the BA intervention on body weight. Additional potential mediators include caloric intake, physical activity and depression (see Table 2 for a complete list of study measures and the timing of these measures).

### Intervention

Both ST and BA conditions will involve an Intensive Treatment and a Maintenance phase. The Intensive Treatment phase will last 6-months and involve 26 sessions. Ten sessions will be 1-hour individual visits, and 16 sessions will be 1.5-hour group behavioral weight loss visits conducted by a registered dietitian or exercise physiologist. The timing of the individual and group visits varies between the two conditions. In the BA condition, participants will begin with 10 weekly individual visits of behavior therapy for treatment of depression, with the initial group behavioral weight loss visits starting on week 9. Participants in the ST condition will begin both the group behavioral weight loss visits and individual health education visits on week 1 (see Tables 3 and 4 for the timing of individual and group sessions in each condition) for two reasons. First, paralleling the BA condition (i.e., first 8-weeks of individual visits of health education attention control only) could lead to differential attrition rates since ST participants may not stay engaged when active treatment is delayed for 8 weeks. Second, this approach is also more consistent with usual care, in which participants would be enrolled and begin treatment immediately.

**Table 3:** Distribution of treatment sessions by condition during the first 12 weeks of the 6-month Intensive Treatment phase.

Week	**1**	**2**	**3**	**4**	**5**	**6**	**7**	**8**	**9**	**10**	**11**	**12**
**ST**	I/G	G	G	G	I/G	G	G	G	G	G	I	G
**BA**	I	I	I	I	I	I	I	I	I/G	I/G	G	G

Note. ST = Standard treatment; BA = Behavioral Activation; I = Individual visit; G = Group visit

**Table 4:** Distribution of treatment sessions by condition during the second 12 weeks of the 6-month Intensive Treatment phase.

Week	**13**	**14**	**15**	**16**	**17**	**18**	**19**	**20**	**21**	**22**	**23**	**24**
**ST**	I	G	I	G	I	G	I	G	I	G	I	I
**BA**	G	G	G	G	G	G	G	G	G	G	G	G

Note. ST = Standard treatment; BA = Behavioral Activation; I = Individual visit; G = Group visit

The 18 month Maintenance phase will consist of 6 monthly 90-minute group sessions and 6 monthly 20-minute counseling phone calls by their therapist or health education counselor, depending on their condition assignment. Then, participants will receive 20-minute counseling phone calls once every 3 months for one year. All visits in both phases will be audiotaped for quality assurance.

### BA Condition

#### Behavioral Weight Loss

Behavioral weight loss groups will be based on the Diabetes Prevention Program (DPP) [24]. A team dietitian will lead 8 of the 16 Intensive Treatment sessions and 3 of the 6 Maintenance sessions. During the Intensive Treatment phase, participants will receive extensive instruction and practice in self-monitoring of caloric and fat intake. Participants will receive calorie goals that are estimated to produce a weight loss of 1–2 pounds per week [i.e., (starting weight × 12) – 500 kcal]. All participants will be given a goal for daily total fat intake in grams that is based on 25% of total calories from total fat. Participants will self-monitor fat and calories daily for 18 weeks and then one week every month for the following 6-months. Participants will be encouraged to work slowly toward regular self-monitoring, beginning with 2–3 days and working up to 5–7 days over the first 16 weeks. Participants who have difficulty tracking their dietary intake and physical activity will be given modest goals with suggestions to increase tracking gradually. Participants will receive assistance to individualize the study diet based on their usual dietary habits. Participants will be given diet diaries for self-monitoring, the "DPP Fat Counter," a guide of nutrition information for hundreds of foods, and multiple resources for obtaining additional information on areas of concern, including eating out and other social activities and recipes. Self-monitoring diaries will be reviewed by the registered dietitian and returned with suggestions for change and positive feedback. Although the original DPP protocol is based on a low-fat diet, this has been updated to include suggestions to decrease detrimental fats preferentially, namely saturated and *trans*-fats, and to substitute whole grains in place of refined carbohydrate foods to match current dietary recommendations put forth by the United States Department of Agriculture Dietary Guidelines for Americans and the American Heart Association Dietary Guidelines [25-27].

The exercise physiologist will lead 8 of the 16 Intensive Treatment group sessions and 3 of the 6 Maintenance sessions. Participants will work toward the goal of 60 minutes of moderate physical activity on 5 days/week. While walking will be encouraged and pedometers provided, participants will also be encouraged to increase their engagement in any of their preferred physical activities. Participants will be lead through a brief moderate physical activity in each session following didactic content. Examples include group walks, pilates, aerobics, and yoga. See Table 5 for the complete list of group topics.

**Table 5:** Content of group sessions during Intensive Treatment (16 sessions) and Maintenance (6 monthly sessions) phases.

**Session #**	**Group Content**
1 D&E	Session 1: Welcome to the Lifestyle Balance Program
2 D	Session 2: Be a Fat Detective
3 E	Session 3: Being Active: A Way of Life (group walk)
4 D	Session 4: Three Ways to Eat Less Fat
5 E	Session 5: Move Those Muscles
6 D	Session 6: Healthy Eating
7 E	Session 7: Creating a Cardiovascular Program; Building Motivation
8 D	Session 8: Taking Charge of What's Around You
9 E	Session 9: Talking Back to Negative Thoughts
10 D	Session 10: Tip the Calorie Balance (group walk)
11 E	Session 11: The Slippery Slope of Lifestyle Change
12 D	Session 12: Problem Solving Diet and Physical Activity
13 E	Session 13: Jump Start Your Activity Plan (group walk)
14 D	Session 14: Four Keys to Healthy Eating Out
15 E	Session 15: You Can Manage Stress
16 E	Session 16: Ways to Stay Motivated
M1 D	Month 1: Food Cues
M2 E	Month 2: Staying Active on Vacation and Holidays (group walk)
M3 D	Month 3: Vitamins and Supplements
M4 E	Month 4: Muscle Training (group practice)
M5 D	Month 5: Food tasting
M6 E	Month 6: Maintaining Motivation After the Program

Note. D = dietitian-led group E = exercise physiologist-led group

#### Behavioral Activation

The treatment protocol for individual sessions is based on brief behavioral activation treatment as developed by Lejuez and colleagues [14]. Each session will emphasize behavioral activation strategies for depression and how the behavioral activation approach can lead to a more enriching active lifestyle (See Table 6 for individual session content) [14,28,29]. A Master's or doctoral level counselor will conduct all 10 of the individual counseling sessions and all 10 of the 20-min telephone sessions. A total of 20 sessions (in person and telephone) are devoted to behavioral activation strategies because efficacy with this protocol has been established in 20–24 sessions [28].

**Table 6:** Content of behavioral activation individual sessions during Intensive Treatment phase.

**Session #**	**Behavioral Activation Content**
1	Getting Started: How depression affects you
2	Create an environment that supports you; behavioral contracting support
3	Activity hierarchies
4	Reviewing Activity Experiments
5	Avoidance As A Habit
6	The Power of ACTION
7	Rumination and Mindfulness
8	Mood Dependence
9	Progress check: Recycle experiments for difficult behaviors
10	Continued Progress: Behavioral Activation on your own

Participants will first be educated about how depression affects them from a behavioral activation perspective. Then, the following behavioral activation skills will be taught: 1) self-monitoring of daily activities and moods; 2) identification of relationships between daily activities and mood; 3) behavioral contracting; 4) identification of values and goals within a variety of life areas including relationships, employment/career, hobbies/recreation, physical/health issues, and spirituality; 5) developing an activity hierarchy in which 15 activities consistent with life goals are rated ranging from "easiest" to "most difficult" to accomplish; 6) regular completion of a master activity log and behavioral checkout to monitor progress as the participant moves through the hierarchy in a progressive manner, moving from the easier behaviors to the more difficult; 7) recognition of avoidance patterns and how they affect mood; and 8) continued assessment of progress and modification of goals as necessary. Earlier sessions will be used to introduce these components and later sessions will include both strategies for increasing success in achieving reasonable life activity goals and for modifying unrealistic or overly difficult activity goals. Behavioral activation for depression is not meant to specifically target weight loss as a goal. Instead, it will address the processes underlying weight dysregulation, including mood regulatory eating, avoidance, and inactivity. Addressing these factors in advance of dietary and physical activity prescriptions may be essential to progress toward weight loss goals during the weight treatment program that follows.

During the Maintenance phase, counselors will contact participants monthly for 20-min phone calls for 6-months and quarterly for one year. As Lejuez and colleagues [14] describe, once participants have progressed to become more skilled at behavioral activation, sessions may become shorter and/or administered by phone. Phone contacts will be used to provide support for continued progress, reinitiate self-monitoring, recycle through the activation process when needed, and keep contact information up-to-date.

### ST Condition

#### Behavioral Weight Loss

Although the timing of the group visits will differ between conditions, the content of the group weight loss intervention in the ST condition will be identical to the BA condition as described above.

#### Attention Control (Health Education)

A health educator with no training in behavior therapy or psychological counseling will conduct all 10 individual sessions during the Intensive Treatment phase and all 20-minute phone calls during the Maintenance phase. Health education sessions were added to the ST condition to serve as an attention control for the nonspecific effects of behavior therapy. An attention control is designed to simulate a behavioral intervention, providing the same amount of contact as the individual counseling in the BA condition, but is not believed to be an effective treatment for depression [30]. This attention control condition lacks components of behavioral activation that are considered integral to its success. The content of the attention control sessions is designed based on prior research demonstrating that provision of information is not sufficient to change behavior [31-37]. Health information about topics relevant to women's health will be discussed. Participants will select from 19 different health education and well-being topics including body composition analyses, menopause, skin health, finances, dressing for different body shapes, and proper footwear (see Table 7 for the full list of topics). To enhance attendance, participants will be informed that the visit will be for a "weigh in" and health education counseling. The health educator will develop a supportive relationship with the participant, provide health education, but will avoid problem solving or counseling with the participant for depression treatment. When participants have specific questions regarding goals or seek advice, they will be referred to their group leader.

**Table 7:** List of topics for standard treatment individual sessions during Intensive Treatment phase.

**Session #**	**Standard Treatment Content**
1	Body Composition Analyses
2	Vitamins for Women: Making sense out of vitamins
3	Menopause: Your survival guide
4	Breast self-exams: Learn how (and why) to perform a breast self-exam regularly
5	Sexual health
6	Skin health: Have your UV photo taken
7	Bone health
8	Footwear for physical activity: Those aching feet
9	Back pain: Oh, my aching back
10	Heartburn and GERD (gastroesophageal reflux disease)
11	Arthritis
12	Alcohol safety: should I be drinking to my health
13	Dementia: Forget where you put your keys again
14	Heart attack: How do I know if I'm having one
15	Home safety
16	Going green
17	What every woman should know about finances
18	Dress for your body type

During the Maintenance phase, health educators will contact participants monthly for 20-minute phone calls for 6 months and quarterly for one year. Calls will be for the purpose of fostering motivation to attend the 1- and 2-year follow-ups as well as to provide educational material on women's health issues.

### Participant Safety

All eligible participants must receive permission from their primary care physician to participate in the study. Depression safety precautions include having participants complete a BDI-II at every individual and group visit, (except when there is an individual and group visit in the same week) to assess depression levels and suicidality. If a participant reports active suicidal ideation with intent, she will be referred for psychiatric treatment and/or escorted to the Emergency Department at UMM. The PI, a licensed clinical psychologist, will review depression severity scores (BDI-II) of all participants on a weekly basis. If a participant's depression symptoms elevate to 30 or higher on the BDI-II (i.e., severe depression) during treatment, alternative treatment will be deemed clinically necessary and that participant will be referred to the appropriate service. Participants who remain depressed (BDI-II > 12) at the end of treatment will receive written materials about depression treatment options and a list of referrals for depression treatment services.

To minimize risks associated with increasing physical activity, intervention personnel will screen participants at each encounter point to assess symptoms that would increase the risk of physical activity and to report concerns as appropriate. In the case of a concern, the study physician and the participant's primary care physician will be consulted. The nutritional adequacy of the diet will be monitored by our senior nutritionist. If there is concern about a participant's nutrient intake, a report is sent to the participant's primary care physician with the area of concern highlighted and appropriate advice provided by our clinical nutritionist.

Safety monitoring procedures are documented in a standard protocol and overseen by the PI and project director. Any adverse events are reviewed by the study physician within 24 hours. If he deems that the adverse event warrants escalation, the data safety monitoring board (DSMB) will then be notified. Unanticipated adverse events or anticipated adverse events that require medical treatment will be reported to the internal review board. All adverse reactions reported by participants will be documented by intervention providers and reviewed on a monthly basis by the PI and project director. Participants will be referred to their healthcare provider for a medical evaluation and follow-up as needed or recommended by the DSMB. The internal review board, the funding agency and the DSMB will be notified of serious adverse events within 48 hours.

### Retention

A participant tracking system will be used to assure that participants are contacted on a timely basis to obtain study data. Participants are also provided a $50 incentive at baseline, 6-months and 1- and 2-year follow-ups for completion of data collection visits. If they are unable to attend, a home visit will be arranged. Following a missed intervention class, participants will receive class materials by mail. Regular telephone contact with individual BA therapists and ST health educators (monthly during the last 6 months of year 1 and every three months during year 2) will help to keep participants engaged in the program and provide an opportunity to obtain updated contact information. If an individual therapist or health educator is concerned about a participant withdrawing, he or she will speak individually with the participant and attempt to address reasons for disengagement or drop-out. For the purposes of retention, at least three alternative contacts, individuals (e.g., family members, friends) who could provide the participant's contact information if it changes, are collected.

### Sample Size Considerations and Statistical Analyses

#### Sample Size

This study will randomize 174 participants into two conditions (87 per condition) who will be followed for 2 years. We expect approximately 90% power for detecting differences in weight between the conditions assuming a weight change difference of 3.1 kg at 1-year (standard deviation = 5.5 kg) and a 25% loss-to-follow-up rate. This projected data for group differences was taken from pilot data at 6 months and projected to one year based on the original DPP weight change data. The standard deviation was a conservative value chosen from a range of successful lifestyle intervention studies [38-40].

### Primary and Secondary Hypotheses-Analysis Plan

The primary outcome compares body weight between the two randomized groups at 6-months and 1-year follow-up using an intent-to-treat analysis. Linear mixed modeling, using SAS PROC MIXED [41] will test time and group interactions to asses whether within-subject change in body weight differs by condition. Analyses will also be conducted to examine whether there is a dose-response relationship between attendance and outcome, where dose is defined in terms of number of sessions attended. In addition, we will model percent change in body weight at 6-, and 12-months using a similar model.

Secondary outcomes will compare the conditions on depression scores, physical activity, daily caloric intake, treatment adherence (session completion), quality of life score, waist circumference, blood pressure, C-reactive protein and serum lipids at 6-months and 1- and 2-years. Analyses for these outcomes will be similar to those conducted for body weight. To explore proposed mechanisms of change, longitudinal associations of body weight and depressive scores, treatment adherence, and physical activity will be modeled using structural equation modeling [42]. Reciprocal influences of body weight and other factors over time will be modeled in structural equation modeling using nonrecursive models [43]. The two conditions will also be compared on body weight and secondary outcomes at the 2-year follow-up relative to baseline to examine long-term maintenance. The analysis for this outcome will be similar to those conducted for body weight at 6 months and 1-year.

### Study Operation and Tracking System

The tracking system used for monitoring participant study activities and providing necessary prompts is based on a communication system using Lotus Notes software R5 from IBM (Release 5.0.11). Because of its responsiveness to changes in study participants' data, the Lotus Notes tracking system functions as a watchdog for the staff, automatically flagging any participant who is nearing a deadline for a measurement or intervention session. This system is ideal for monitoring the progress of study participants and interventionists, and alerts the project manager and other study personnel to all study data collection points. The software also will be used to gather and store data that can be shared by individuals both on-site and in remote locations. This includes 24 hour dietary and physical activity recall call assignments and the status of these and other data which are collected at remote sites and downloaded directly to the Lotus Notes system. It also provides reports of data such as those from participant questionnaires and blood test results. Storage of such data provides instant access to critical information for intervention and data collection. Multiple levels of password protection are utilized to ensure data security.

### Data Management

All study data are entered into computerized data files utilizing: 1) Lotus Notes for participant tracking and intervention data entry, 2) the Nutrition Data System for Research software computer-assisted telephone interview system for 24 hour recalls, and 3) Snap Surveys for double-entry verification of data from paper and pencil forms. Data sets will be cleaned, verified and archived, and then read into SAS (version 9.1) data sets, which also are archived. All analytic and tracking database files are stored in a secure network drive and backed up at daily basis. On a weekly, monthly, quarterly and yearly basis complete backups are made of all database files. One copy is saved on-site and one off-site. Separate archival databases are permanently maintained.

At the time of data collection, research assistants will review participant responses on questionnaires with participants present. Skipped or incorrectly addressed items will be brought to the participant to correct. Data from pencil and paper forms are entered using a double entry format. All of the data entry systems employ automatic checks for values that are out of range or represent errors of logic. These procedures reduce transcription errors for hard copy data to close to zero (<0.5%). A random 10% of all 24 hour recall telephone interviews will be monitored. Monthly exploratory analyses will be conducted to detect outliers [44].

### Treatment Fidelity

#### Individual Sessions (Behavioral Activation and Health Education)

Treatment fidelity checklists (2 Provider Checklists and 1 Auditor Checklist) were developed to monitor treatment fidelity. A set of Provider Checklists were developed for each condition. Interventionists will complete a Provider Checklist after each session. A random selection stratified by counselor of 10% of audio-recorded individual sessions will be reviewed and an Auditor Checklist completed by individuals trained in behavioral activation. The Auditor Checklist covers treatment objectives from both health education and behavior therapy for that session. Perfect treatment fidelity (and zero contamination) for a health education session will be evidenced by 100% of health education objectives and 0% of behavior therapy objectives met. When a session is reviewed with less than 85% of treatment-specific objectives met and/or any evidence of contamination (>0% other condition objectives met), the auditor will inform the PI who will remediate interventionist training as needed. This process will continue through all treatment waves so that counselor/educator drift can be swiftly corrected.

#### Treatment Fidelity of Group Weight Loss Sessions

Provider and auditor checklists were created for the group weight loss visits to ensure that treatment objectives for each group session are met in each condition. Contamination will be evident in group sessions if elements of behavioral activation are being met during group sessions. Group leaders will not be trained in or have expertise with behavioral activation or any other therapeutic approaches for depression. However, participants with depression may spontaneously bring depression into the group discussion. Group leaders will emphasize that the purpose of the groups is to help participants improve their lifestyle habits, which can have a positive effect on stress and depression. Group leaders will be trained to stay within the protocol and not engage in problem-solving around depression-specific issues. They also will notify the PI if a participant requests further help, to determine whether a referral is deemed necessary. As for individual visits, an audited session with less than 85% of treatment-specific objectives met and/or any evidence of contamination will be brought to the attention of the PI who will remediate interventionist training as needed.

## Discussion

The present study aims to test whether sequencing behavioral treatment for depression and behavioral weight loss treatment produces greater weight loss than administering standard weight loss treatment alone in depressed, obese women. Research suggests that depressed individuals tend to fair worse in weight loss interventions than their non-depressed counterparts, perhaps because their depression remains untreated and presents a barrier to behavior change [2]. Behavioral activation involves increasing activity, decreasing avoidance behaviors and changing maladaptive coping strategies, all of which might facilitate the dual goals of decreasing depression and increasing weight loss.

Behavioral activation, cognitive-behavioral therapy, and cognitive therapy were considered for combining with weight loss treatment, but behavioral activation was selected for several reasons. First, behavioral activation is briefer than cognitive behavioral therapy [45] with efficacy in as little as 8 sessions [29]. Second, behavioral activation is relatively uncomplicated compared to cognitive therapies and so can be used with participants with varying levels of insight and intellectual capacity. Third, the intense focus on increasing *activity *is appropriate to the goals of treatment for both depression and weight loss. The goal of behavioral activation is to substitute adaptive coping behaviors for maladaptive ones. Weight loss could be facilitated by helping the participant increase her arsenal of mood regulatory behaviors to break an overreliance on eating for coping with negative moods/boredom and to gradually adopt more physically active coping behaviors. Fourth, the focus on eliminating avoidance in behavioral activation is relevant to both mood and weight. Avoidance among obese participants can take the form of excessive eating and prolonged sedentary activities (e.g., hypersomnia, TV, computer/internet use) and prevent the participant from engaging in other more reinforcing and active aspects of life.

Multiple behavior change is touted as an economical and efficient way of reducing health risk [51], however the necessity of combining treatment for depression and obesity goes beyond economy and efficiency. The processes maintaining the two disorders when they co-occur may be so intertwined that improvements in one condition may be contingent on improvements in the other. While combining weight loss and depression therapies may seem warranted, administering two fairly intensive treatments simultaneously may be too overwhelming for patients who are physically and emotionally taxed. Preliminary data revealed that a simultaneous approach is feasible, strongly impacts depression, but may not lead to greater weight loss compared to a weight loss intervention that does not address depression [19]. The impact of weight loss counseling might be improved if administered once depressive symptoms have begun to subside. As such, depression treatment will be administered prior to intensive efforts towards weight loss. This approach may not only be more clinically indicated, but also more cost-effective since clinical efforts towards weight loss will be administered during a "window of opportunity" when the patient is better prepared to respond.

There are some limitations of the study. The intensity of the treatment protocol may make adoption in practice settings with limited resources difficult. However, since treatment for depression is usually a reimbursable service, but weight loss treatment is not, packaging depression and weight loss treatment together may be a more affordable option for many patients. Also, exclusion criteria limit generalizability to at-risk populations such as people with type 2 diabetes, severe depression, or people who are on certain medications associated with weight gain. Once efficacy is established, effectiveness studies with less stringent exclusion criteria are needed to evaluate the impact in real world settings.

## Conclusion

While evidence-based treatments exist for both obesity and depression separately, these disorders are frequently comorbid and treatment approaches have not been established for these disorders when they co-occur. This trial attempts to promote weight loss in depressed, obese women by administering behavioral activation treatment for depression directly prior to an evidence-based weight loss treatment. Given the high rates of psychological disorders among individuals with obesity, research is needed to understand the mechanisms linking psychological disorders with obesity, as well as whether weight loss treatment approaches that are tailored to the psychological disorder improve both weight loss and mental health outcomes.

## Competing interests

The authors declare that they have no competing interests.

## Authors' contributions

SP conceived, designed and obtained funding for the study. YM, BO, PM, JB and SC participated in the study's design. JO and PM are involved in the study's coordination. YM, SC and KS participate in the study's data management. IO is involved in participant safety and consultation. KS drafted the manuscript. All authors read and approved the final manuscript.

## Pre-publication history

The pre-publication history for this paper can be accessed here:


